# Assessment of Alzheimer’s disease-related biomarkers in patients with obstructive sleep apnea: A systematic review and meta-analysis

**DOI:** 10.3389/fnagi.2022.902408

**Published:** 2022-10-13

**Authors:** Wenqi Cui, Zhenghao Duan, Zijian Li, Juan Feng

**Affiliations:** Department of Neurology, Shengjing Hospital of China Medical University, Shenyang, China

**Keywords:** obstructive sleep apnea, Alzheimer’s disease, amyloid-β 42 (Aβ42), total tau, phosphorylated tau (p-tau), PET scans

## Abstract

Increasing evidence links Alzheimer’s disease (AD) to various sleep disorders, including obstructive sleep apnea (OSA). The core AD cerebrospinal fluid (CSF) biomarkers, including amyloid-β 42 (Aβ42), total tau (t-tau), and phosphorylated tau (p-tau), can reflect key elements of AD pathophysiology before the emergence of symptoms. Besides, the amyloid-β (Aβ) and tau burden can also be tested by positron emission tomography (PET) scans. Electronic databases (PubMed, Embase, Web of Science, and The Cochrane Library) were searched until August 2022 to assess the AD-related biomarkers measured by PET scans and CSF in OSA patients. The overall analysis showed significant differences in Aβ42 levels (SMD = −0.93, 95% CI:−1.57 to −0.29, *P* < 0.001) and total tau (t-tau) levels (SMD = 0.24, 95% CI: 0.01–0.48, *P* = 0.308) of CSF, and Aβ burden (SMD = 0.37, 95% CI = 0.13–0.61, *P* = 0.69) tested by PET scans between the OSA and controls. Furthermore, CSF Aβ42 levels showed significant differences in patients with moderate/severe OSA compared with healthy control, and levels of CSF Aβ42 showed differences in OSA patients with normal cognition as well. Besides, age and BMI have influences on heterogeneity. Our meta-analysis indicated abnormal AD-related biomarkers (CSF and PET scans) in patients with OSA, supporting the current hypothesis that OSA, especially moderate/severe OSA, may start the AD neuropathological process.

**Systematic review registration:**

[https://www.crd.york.ac.uk/PROSPERO/], identifier [CRD42021289559].

## Introduction

Obstructive sleep apnea (OSA), characterized by repetitive upper airway collapse during sleep, is a common, chronic sleep-related breathing disorder that results in temporarily stop or decrease breathing during sleep, leading to symptoms such as snoring during sleep, morning headache, daytime sleepiness, and fatigue. Severity is conventionally graded by the Apnea/Hypopnea Index (AHI) (Semelka et al., 2016; Chang et al., 2020). As estimated, nearly 1 billion adults aged 30–69 years worldwide suffered from OSA, and almost 425 million people with moderate to severe OSA (Benjafield et al., 2019). Recently, increasing evidence suggests that OSA is not only a frequent diagnosis in Alzheimer’s disease (AD) patients but also raises the risk of developing MCI and AD, indicating that OSA may also be a risk factor for AD neurodegeneration (Lim and Pack, 2014; Liguori et al., 2017). However, in contrast to other proven risk factors for the development of AD, symptoms of OSA can be significantly improved in clinical practice (Fernandes et al., 2022). Thus, it is necessary to figure out whether OSA may promote AD pathology.

Dementia is an age-related, chronic, and progressive disorder, which is characterized by progressive cognitive impairment sufficient to impact activities of daily living (Shamseer et al., 2016; Lane et al., 2018). As the most prevalent form of dementia, AD is pathologically characterized by the abnormal accumulation of Aβ and intracellular neurofibrillary tangles (NFT) from hyperphosphorylation of tau. The clinical symptoms of AD are preceded in many cases by a period of mild cognitive impairment (MCI) (Lu et al., 2018). Nevertheless, in a significant proportion of young-onset AD cases, symptoms are atypical, which often cause late diagnosis, thus biomarkers of AD are needed for early and exact diagnosis (Graff-Radford et al., 2021). Current evidence suggests that cognitive decline in AD is associated with a decrease in Aβ42, an increase in t-tau and phosphorylated tau (p-tau) levels in CSF, and an increase in amyloid PET uptake (Kandimalla et al., 2011; Olsson et al., 2016). Moreover, MRI biomarkers, such as gray matter atrophy and functional brain connectivity, and blood–brain barrier (BBB) also emerged as markers of AD (Dennis and Thompson, 2014; Ishii and Iadecola, 2020; Nakazawa et al., 2022). The deposition of Aβ and NFTs formed by tau in the brain cortex can be detected by a PET scan and begins many years before the onset of symptoms reaching a plateau by the time the first cognitive impairments become clear (Valotassiou et al., 2018). In both MCI and AD patients, increased [^11^C] and [^18^F] florbetaben uptake for Aβ burden and PiB 18F-AV1451 uptake for tau protein burden have been reported (Rabinovici et al., 2007; Pontecorvo et al., 2017). Therefore, the alterations of CSF and PET biomarkers may reflect AD pathology years before cognitive symptoms, and thus are useful in predicting the future decline and providing a critical stage for potential interventions, as tissue damage is presumably mild (Osorio et al., 2014).

Currently, several efforts have been made to better understand the risk of sleep-disordered breathing (SDB), especially OSA, as a potential contributor to AD (Liguori et al., 2017; Sharma et al., 2018; Jorge et al., 2020; Diáz-Román et al., 2021), while some studies questioned the link between OSA and AD (Blackwell et al., 2015; Mohajer et al., 2020). Evidence indicated that no associations were observed between the SDB parameters and clinically significant cognitive decline (Blackwell et al., 2015; Jorge et al., 2020). Also, studies revealed that no diagnostic-dependent association of SDB with either gray matter atrophy or brain aging was measured by MRI (Mohajer et al., 2020). Thus, we evaluated CSF and PET biomarkers, changes in them may appear to precede cognition decline and gray matter atrophy, to better assess the role of OSA in the AD pathological process.

## Data source and search

Systematic electronic databases (PubMed, Embase, Web of Science, and The Cochrane Library) were searched for articles published in English from their inception until August 2022 with the following search items: “OSA” OR “sleep disordered breathing” AND “amyloid” OR “tau” OR “CSF” OR “PET” (Appendix A). Filters were applied to limit research to human studies in the English language. The reference lists from relevant original and review articles of the eligible publications were searched manually for additional studies and were assessed in accordance with the selection criteria. Citations and abstracts of all the studies have been checked to prevent duplications.

## Study selection

This systematic review was conducted according to the PRISMA guidelines and followed a predetermined protocol (PROSPERO No. CRD42021289559) (Page et al., 2021). The selection criteria of the studies were as follows: (1) studies that included patients with a diagnosis of OSA (mild OSA or moderate/severe OSA) and a control group; (2) studies that contained CSF biomarkers (Aβ42 or tau) or PET scans (Aβ and tau burden); (3) studies that made data available in the publication or *via* contact with the authors to allow computation of correlation coefficients.

**FIGURE 1:**
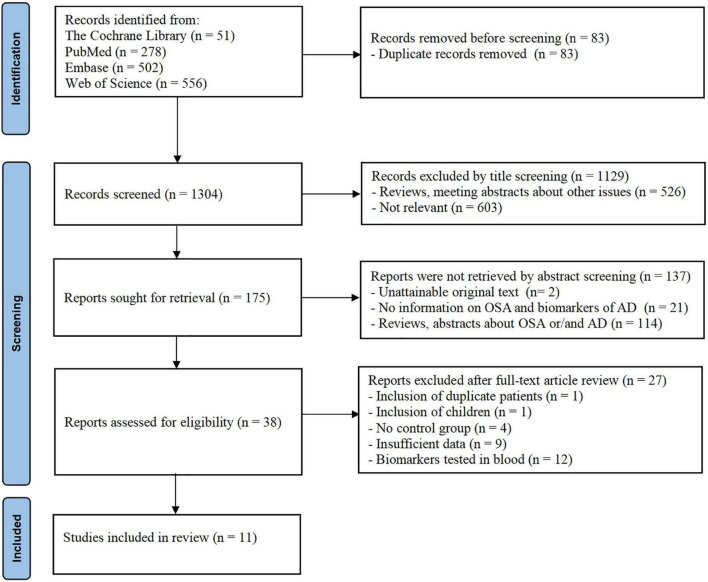
Flowchart of search strategy and study selection.

The title and abstract of the article were screened to identify whether the study fulfilled the inclusion criteria. The initially selected articles were retrieved for full text by two independent reviewers to establish the final eligibility of the articles. Disagreements were adjudicated by a third reviewer based on the full text. A summary of the methodologies and main findings of the 11 studies is presented in Figure 1.

## Diagnosis and measurement of obstructive sleep apnea

In all the included studies, OSA was determined by nocturnal polysomnographic (PSG) recording, which was performed according to the American Academy of Sleep Medicine (AASM) criteria (Duce et al., 2015). OSA with AHI, higher than 5 events per hour and less than 15 events per hour, is defined as mild OSA. AHI of 15 events per hour or higher was applied to establish a diagnosis of moderate to severe OSA (Mysliwiec et al., 2020). We included studies that evaluated OSA by polysomnography respiratory monitoring (PSG) or home sleep monitoring.

## Diagnosis of mild cognitive impairment/subjective cognitive impairment and measurement of cerebrospinal fluid biomarkers

In the studies we included, MCI was defined by the NIA-AA workgroup (Jack et al., 2018). Subjective cognitive impairment (SCI) was defined as subjective memory deficits reflecting a decline over the previous 5 or 10 years, absence of overt cognitive deficits, and cognitive performance in a general normal range (Rabin et al., 2017).

In included studies, CSF biomarker levels (Aβ42, t-tau, and p-tau) were measured by the ELISA method. Aβ burden was tested by PET scans (including ^11^C PIB, 18F-florbetapir/florbetaben, and 18F-NAV4694). For studies including PET imaging, the PET SUVR was used as a continuous variable to explore the Aβ burden in OSA (Valotassiou et al., 2018).

## Data extraction and quality assessment

We used a standardized table to extract the following information from all included articles: first author’s name, publication year, the study design, sample size, age, gender, types of AD-biomarkers, and cognition status.

The quality of included studies was assessed using the Newcastle Ottawa Scale (NOS) (Appendix B) as recommended by the Cochrane Non-Randomized Studies Methods Working Group (Stang, 2010). In this scale, each study is evaluated according to eight items categorized into three groups: sample selection, comparability, and exposure. The total score ranged from 0 to 9 points with higher scores indicating higher quality (7 ≤ score ≤ 9 indicates high quality; 4 ≤ score ≤ 6 indicates medium quality; score ≤ 3 indicates low quality) (Lo et al., 2014). Quality assessment was performed according to the NOS by two authors independently. Discrepancies in the score were resolved through discussion by the authors.

## Statistical analysis

The meta-analysis was performed by using the STATA statistical software 15.0 (Cochrane Collaboration, Oxford, UK). The standard mean differences (SMD) and corresponding 95% confidence intervals (CI) were pooled to assess AD biomarkers (CSF and PET scans). Effect sizes with a *P*-value of <0.05 were considered significant (Egger et al., 1997). Heterogeneity across studies was assessed according to *I*^2^ statistics. Values of 25, 50, and 75% are considered as low, moderate, and high heterogeneity, respectively (Higgins et al., 2003). A fixed-effect model was selected if *I*^2^ < 50%. Otherwise, a random-effect model was selected. The subgroup analysis was conducted using age, BMI, cognition status, and severity of OSA. Finally, we quantitatively assessed publication bias using Begg’s adjusted rank test (Begg and Mazumdar, 1994).

## Results

### Description of studies

In total, 1,304 records were identified from the database searches. After screening the titles and abstracts, 38 studies were reviewed for further assessment. Additionally, one study with duplicate involved patients, one study only included children, four studies without a control group, nine studies with insufficient data were excluded, and 12 studies that tested biomarkers in serum or plasma were excluded. As a result, 11 eligible studies were included in the final meta-analysis. Figure 1 shows the flow chart of the study selection process in this meta-analysis.

The characteristics of the included studies are shown in Table 1. Nine studies were cross-sectional and two were case-control studies. Demographic variables such as age, sex, and education were adjusted statistically in most of the studies. All these studies were identified to be of high or moderate quality using the NOS. The details of the NOS for each study are shown in Appendix B.

**TABLE 1:** Extracted and summarized details on subjects, methods, and measurements of the included studies.

Study	Total *n*	Design	% F	Age	Type	Method	Cognition
Osorio et al., 2014	Mild OSA *n* = 51	CS	58.8	67.8 ± 7	CSF: Aβ42	ELISA	CN
	Moderate/serve OSA *n* = 19		57.9	70.1 ± 8.8	t-ta u		
	Control *n* = 25		68	65.3 ± 8.2	p-tau		
Ju et al., 2016	Moderate/serve OSA *n* = 10	CS	30	56.4 ± 4	CSF: Aβ42	ELISA	CN
	Control *n* = 31		48	53.2 ± 5.7	t-tau		
					p-tau		
Liguori et al., 2017	Moderate/serve OSA *n* = 25	CS	68	67.96 ± 7.92	CSF: Aβ42	ELISA	SCI
	Control *n* = 15		60	66.6 ± 7.10	t-tau		
					p-tau		
Yun et al., 2017	Moderate/serve OSA *n* = 19	CS	52.6	56.7 ± 3.8	PET: Aβ burden	^11^C-PiB	CN
	Control *n* = 19		52.6	56.7 ± 4.2			
Sharma et al., 2018	Mild OSA *n* = 43	CS	57.9	68.6 ± 7.19	CSF: Aβ42	ELISA	CN
	Moderate/serve OSA *n* = 15		51.4	70.68 ± 7.69			
	Control *n* = 50		69.1	67.56 ± 7.32			
Elias et al., 2018	OSA *n* = 42	CS	0	67.69 ± 5.37	PET: Aβ burden	^18^F-florbetaben	−
	Control *n* = 77		0	68.30 ± 3.86	tau burden	^18^F-AV1451	
Liguori et al., 2019	Moderate/serve OSA *n* = 20	CS	70	58.75 ± 3.53	CSF: Aβ42	ELISA	CN
	Control *n* = 15		53.3	63.8 ± 8.46	t-tau		
					p-tau		
Jackson et al., 2020	OSA *n* = 34	CC	44.1	57.8 ± 8.5	PET: Aβ burden	^18^F-NAV4694	−
	Control *n* = 12		50	57.1 ± 8.2			
André et al., 2020	Moderate/serve OSA *n* = 20	CC	58.3	69 ± 3.98	PET: Aβ burden	^18^F-Florbetapir	−
	Control *n* = 15		77.4	69.19 ± 3.53			
Diáz-Román et al., 2021	Mild OSA *n* = 11	CS	−	−	CSF: Aβ42	ELISA	MCI
	Moderate/serve OSA *n* = 33				t-tau		
	Control *n* = 25				p-tau		
Fernandes et al., 2022	Moderate/serve OSA *n* = 34	CC	20.6	68.44 ± 9.98	CSF: Aβ42	ELISA	CN
	Control *n* = 34		20.6	69.02 ± 6.08	t-tau		
					p-tau		

OSA, obstructive sleep apnea. Design: CS, cross-sectional; CC, case-control study; F, females; Type: Aβ42, amyloid-β 42; t-tau, total tau; p-tau, phosphorylated tau; CSF, cerebrospinal fluid; PET, positron emission tomography. Method: enzyme-linked immunosorbent assays. Cognition: CN, cognitively normal; MCI, mild cognitive impairment; SCI, subjective cognitive impairment.

### Abnormal findings of cerebrospinal fluid biomarkers in obstructive sleep apnea

**FIGURE 2:**
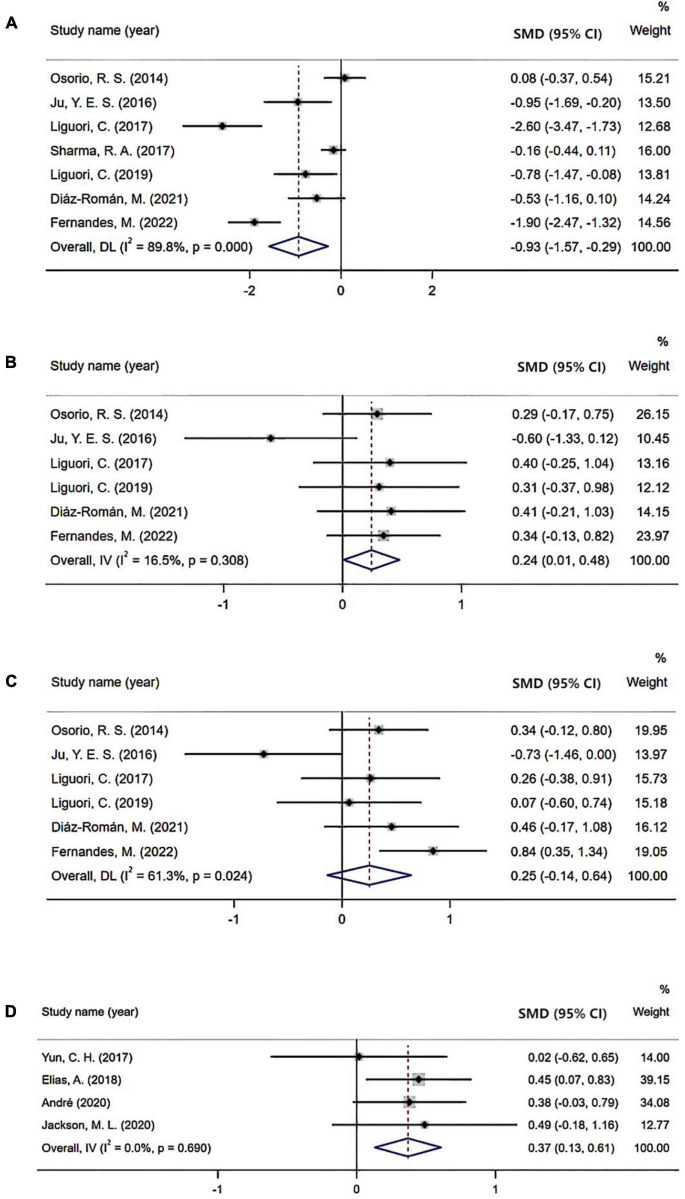
Forest plot comparing levels of cerebrospinal fluid (CSF) and positron emission tomography (PET) biomarkers in subjects with obstructive sleep apnea (OSA) and controls: **(A)** CSF Aβ42 level; **(B)** CSF t-tau level; **(C)** CSF p-tau level; **(D)** PET Aβ burden.

Seven studies investigated Aβ42 levels in CSF enrolling 314 patients with OSA and 230 controls. Results demonstrated that patients with OSA had lower Aβ42 levels in the CSF compared with controls, with an effect size of −0.93 (95% CI: −1.57 to −0.29, *P* < 0.001; Figure 2).

Six studies evaluated t-tau levels in CSF including 203 OSA subjects and 133 controls. In the overall analysis, an increased level of t-tau was observed in patients with OSA (SMD = 0.24; 95% CI = 0.01–0.48; *P* = 0.308; Figure 2).

Six studies reported p-tau levels in the CSF enrolling 203 OSA and 133 control subjects. However, there was no significant difference in the level of CSF p-tau between the two groups (SMD = 0.25; 95% CI: −0.14 to 0.64, *P* = 0.024; Figure 2).

**FIGURE 3:**
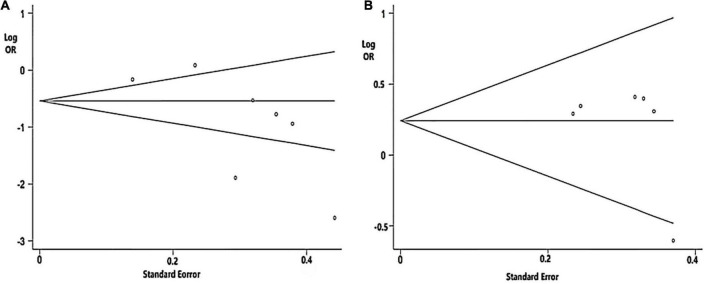
Funnel plot analysis used to detect publication bias: **(A)** CSF Aβ42 level (*P* = 0.072 in Begg’s test); **(B)** Cerebrospinal fluid (CSF) t-tau level (*P* = 0.452 in Begg’s test).

Begg’s tests of Aβ42 and t-tau were not significant (Figure 3).

### Abnormal amyloid-β burden tested by positron emission tomography scans

Four studies evaluated the Aβ burden tested by PET imaging. In these studies, higher Aβ burden was observed in patients with OSA than in control cohorts (SMD = 0.37; 95% CI = 0.13–0.61, *I*^2^ = 0%, *P* = 0.69; Figure 2).

### Subgroup analysis

**FIGURE 4:**
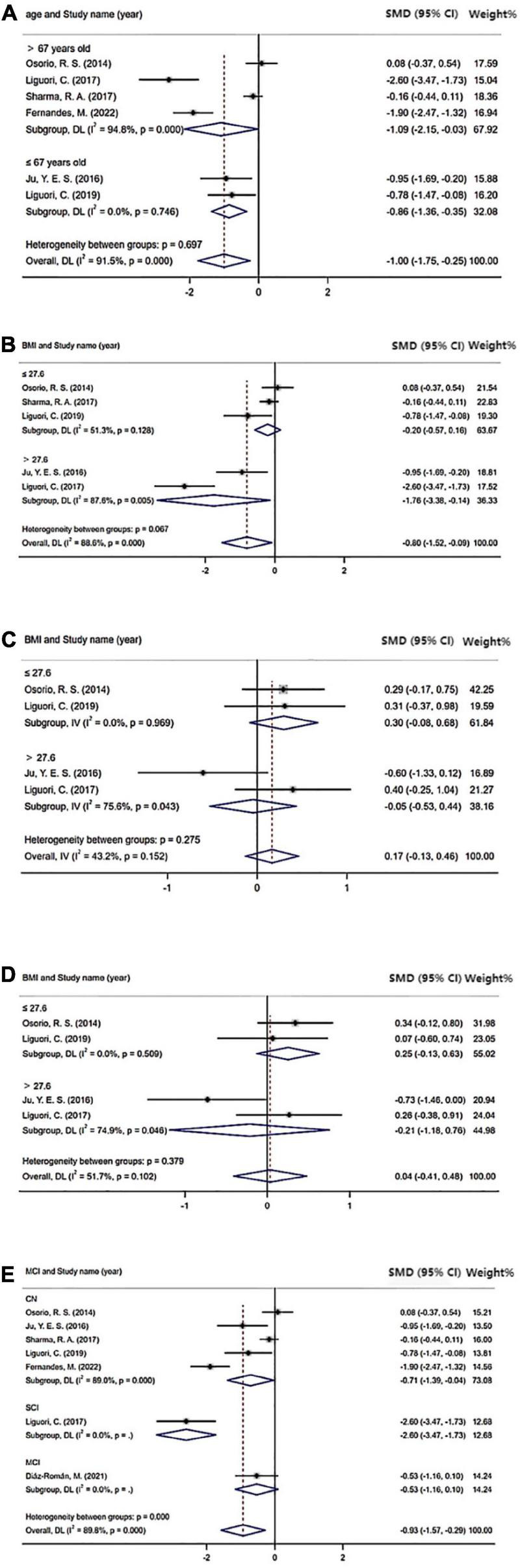
Sub-group analysis: **(A)** Cerebrospinal fluid (CSF) Aβ42 level based on age; **(B)** CSF Aβ42 level based on BMI; **(C)** CSF t-tau level based on BMI; **(D)** CSF p-tau level based on BMI; and **(E)** CSF Aβ42 level based on cognition status.

The results of the subgroup analysis, which explored the potential sources of heterogeneity, are summarized in Figure 4. Subgroups were stratified by age (≤67 years old or >67 years old), BMI (≤27.6 or >27.6), cognition status (normal cognition, MCI, or SCI), and severity of OSA (mild vs. normal, moderate/severe vs. normal, and mild vs. moderate/severe). The cutoff points of age and BMI were determined by the mean value of all included studies. The pooled effects of the Aβ42 levels in CSF across studies that included patients >67 years old was significant (Aβ42: SMD = −1.09; 95% CI = −2.15 to −0.03; *I*^2^ = 94.8%, *P* < 0.001) compared with non-significant correlations found in studies that included patients ≤ 67 years old (Aβ42: SMD = −0.86; 95% CI = −1.36 to −0.35; *I*^2^ = 0.0%, *P* = 0.746). Furthermore, in studies involving patients with normal cognition, levels of Aβ42 in CSF also showed significant difference in OSA patients compared with control (Aβ42: SMD = −0.71; 95% CI = −1.39 to −0.04; *I*^2^ = 89%, *P* < 0.001). Besides, no significant differences in t-tau and p-tau levels were found in OSA patients in age and cognition subgroups. In the subgroup analysis based on BMI, the pooled effects of the biomarker levels in CSF across studies that included patients with BMI >27.6 were significant (Aβ42: SMD = −1.76; 95% CI = −3.38 to −0.14; *I*^2^ = 87.6%; *P* < 0.001; t-tau: SMD = −0.05; 95% CI = −0.53 to 0.44; *I*^2^ = 75.6%; *P* = 0.043; p-tau: SMD = −0.21; 95% CI = −1.18 to 0.76; *I*^2^ = 74.9%; *P* = 0.046) compared with non-significant correlations found from studies that included patients with BMI ≤ 27.6 (Aβ42: SMD = −0.20; 95% CI = −0.57 to 0.16; *I*^2^ = 51.3%, *P* = 0.128; t-tau: SMD = 0.30; 95% CI = −0.08 to 0.68; *I*^2^ = 0.0%; *P* = 0.969; p-tau: SMD = 0.25; 95% CI = −0.13 to 0.63; *I*^2^ = 0.0%; *P* = 0.509).

**FIGURE 5:**
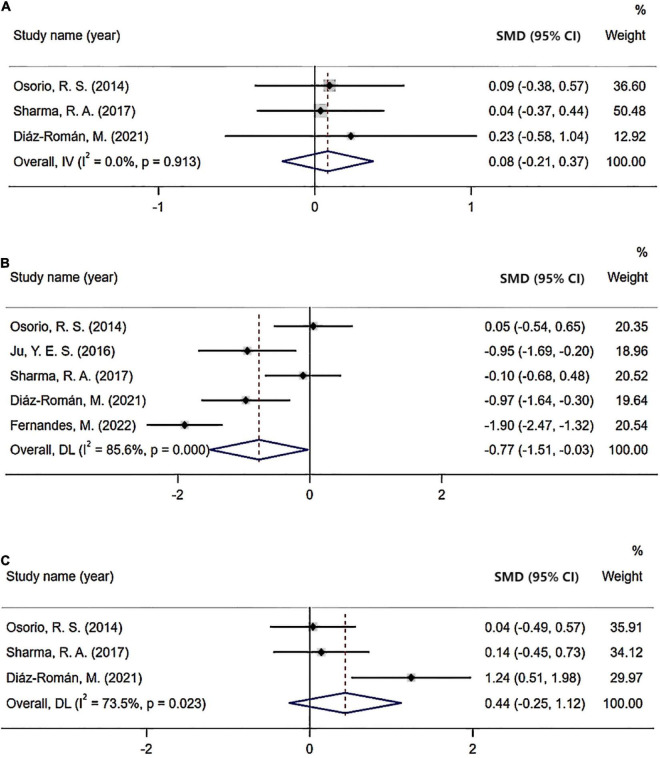
Forest plot of cerebrospinal fluid (CSF) Aβ42 level: **(A)** Mild obstructive sleep apnea (OSA) compared with healthy control; **(B)** Moderate/severe OSA compared with healthy control; **(C)** Mild OSA compared with moderate/severe OSA.

As for subgroup analysis of the severity of OSA, CSF levels of Aβ42 were significant in patients with moderated and severe OSA (Aβ42: SMD = −0.71; 95% CI = −1.39 to −0.04; *I*^2^ = 89%, *P* < 0.001; Figure 5) compared with healthy control. However, CSF levels of t-tau and p-tau were not significant in patients with moderated and severe OSA compared with the control. Also, there were no significant difference in CSF levels of Aβ42, t-tau, and p-tau in both the mild OSA groups compared with the healthy control and mild OSA groups compared with moderated and severe OSA group.

## Discussion

This meta-analysis explored the abnormal Aβ and tau burden in OSA patients *via* PET scans or CSF evaluation and suggested that OSA, especially moderate/severe OSA, possibly started the AD neuropathological process. Our analysis showed decreased Aβ42 level and increased t-tau level in the CSF, and an increased Aβ burden tested by PET imaging in OSA patients compared with control subjects. Besides, moderate/severe OSA, rather than mild OSA, showed a more significant difference in AD-related biomarkers of CSF compared with healthy control. The decrease of Aβ42 was more significant in older OSA patients and even with normal cognition. Moreover, OSA patients with a higher value of BMI showed a more significant difference in CSF Aβ42 and t-tau levels. According to a recent hypothesis, while OSA could be a consequence of events in the progression of AD pathology, alternatively, OSA may precipitate AD pathogenesis. The latter hypothesis would present an exciting opportunity to slow AD pathology with sleep interventions (Sharma et al., 2018). Our study may reinforce the latter hypothesis of the AD neurogenerative process triggered by OSA.

The Aβ42 contributes to cerebral amyloid angiopathy in AD and decreased concentrations of Aβ42 in CSF have all proven to be useful in the diagnosis of AD (McKhann et al., 2011). Currently, increasing studies have linked OSA to decreased concentrations of Aβ42 in CSF (Ju et al., 2016; Liguori et al., 2017, 2019; Sharma et al., 2018; Diáz-Román et al., 2021; Fernandes et al., 2022). However, there were also studies indicating no significant difference of Aβ42 levels in CSF of OSA patients (Osorio et al., 2014; Diáz-Román et al., 2021). Our results were in line with past studies indicating decreased concentrations of CSF Aβ42 level in OSA even in patients with normal cognition, which could indicate the role of OSA in the start in AD pathogenesis (Liguori et al., 2017; Bubu et al., 2019). We also stratified BMI in OSA patients and found BMI may influence the CSF Aβ42 level in OSA patients, which is a controllable factor for OSA patients to delay AD progression.

Increased levels of p-tau and t-tau in CSF could reflect cortical tangle formation and axonal degeneration or injury and can be used as a general marker in AD (Kazim et al., 2022). Many studies have reported increased CSF t-tau and p-tau levels in OSA patients (Diáz-Román et al., 2021; Fernandes et al., 2022). Nevertheless, several studies show no significant difference in CSF t-tau and p-tau levels in OSA patients (Osorio et al., 2014; Ju et al., 2016; Liguori et al., 2017, 2019). We found increased levels of CSF t-tau in OSA patients compared with the control. In contrast with Aβ42 and t-tau levels, we did not find a significant difference in concentrations of CSF p-tau levels in OSA patients. The results may be attributed to the fact that patients in the enrolled study were with normal cognition or MCI. While some studies observed an increase in tau levels in both NC and MCI patients with OSA compared to non-OSA individuals, we could not find differences in relation to p-tau in OSA patients at an early stage of AD compared with control (Liguori et al., 2017; Bubu et al., 2019; Diáz-Román et al., 2021). Furthermore, we found OSA patients with larger BMI may have increased CSF t-tau and p-tau levels than OSA patients with lower BMI.

*In vivo* imaging with PET provides a unique opportunity to quantify Aβ and tau burden and examine their direct associations with sleep outcomes. We analyzed the Aβ burden tested by PET scans in OSA patients. We found significant differences in Aβ burden tested by PET imaging between OSA patients and the control group in line with previous studies (Yun et al., 2017; André et al., 2020). Results suggested that OSA could accelerate amyloid deposition and may contribute to the development or progression of AD. More research on the relationship between PET scans and OSA is needed to be conducted to better explain the mechanism.

Increasing evidence reported severity of the OSA, indexed using the AHI, was associated with Aβ42, t-tau, and p-tau levels (Sharma et al., 2018; Diáz-Román et al., 2021). In our analysis, CSF levels of Aβ42 were significant in patients with moderated and severe OSA compared with healthy control, while no significant differences were found in both mild OSA group compared with healthy control and mild OSA group compared with moderated and severe OSA groups. It indicated that changes in CSF biomarkers are more pronounced in moderate and severe OSA groups, which implies that existing therapies reducing the severity of OSA could delay the progression to MCI or dementia in OSA, especially in moderate and severe OSA patients (Sharma et al., 2018).

Although the mechanism of the relationship between OSA and AD is still not clear, studies have proposed several pathways through which OSA may contribute to increased AD biomarkers in the brain. Currently, the function of BBB has been introduced to the association between OSA and AD (Lim and Pack, 2014). Intermittent hypoxia generated by OSA may cause the dysfunction of the BBB, leading to an increase in membrane permeability, and eventually dysfunction or degeneration of neuronal (Kerner and Roose, 2016). Moreover, the permeability of the BBB can also be altered by influencing a variety of procoagulant factors, increasing oxidative stress, and inducing chronic inflammation by OSA (Lim and Pack, 2014; Zolotoff et al., 2021). Another possible mechanism through which OSA might increase amyloid deposition is influencing CSF-interstitial fluid (ISF) exchange promoted by the glymphatic system resulting in decreased clearance of ISF Aβ42. During obstructive apnea, respiratory effort against a closed airway creates elevated intrathoracic and intracranial pressure and a sudden pressure reversal at the end of the apnea. Repetitive high-pressure fluctuations may impede the glymphatic flow of metabolites from ISF into CSF. This would result in decreased clearance of ISF Aβ42 and increased amyloid deposition. Moreover, nocturnal intermittent hypoxia damages enzymes susceptibility to hypoxia and is responsible for the β-amyloid metabolism, which can result in amyloid deposition (Ju et al., 2016; Liguori et al., 2017; Sharma et al., 2018). Besides, sleep deprivation results in a significant increase in tau pathology, and it is a chronic stressor inducing the development of AD neurodegeneration (Liguori et al., 2017). As for the sleep aspect, OSA leads to decreased slow wave activity (SWA), increased synaptic activity, decreased glymphatic clearance, and increased Aβ (Osorio et al., 2014).

Heterogeneity was also analyzed in our study. According to our overall analysis, the heterogeneity of CSF levels of Aβ42 was large, whereas the difference in the t-tau, p-tau, and Aβ burden in PET scan in OSA compared with control was not significant. Our subgroup analysis indicated that age, BMI, AHI, and cognition status may be influencing factors that could be partly responsible for heterogeneity.

Our study has several limitations that warrant consideration. First, the included studies exploring the relationship between AD biomarkers and OSA had relatively small numbers of subjects. Due to the small number of original studies and their relatively small patient cohorts, we could not analyze the performance with different age, BMI, AHI, or cognition status of Aβ and tau burden by PET scans. Also, the results in OSA patients with the MCI subgroup could not be concluded in our analysis due to insufficient data. Second, although we tried to explore the source of heterogeneity by subgroup analysis in our study, there were still some factors that could not be assessed by our result for example gender, APOE gene, and more sleep parameters (Diáz-Román et al., 2021). Furthermore, we could not evaluate whether existing therapies for OSA could improve the AD-related biomarkers from involved studies. More longitudinal epidemiological studies are needed to address the role of OSA in AD pathology.

## Conclusion

In conclusion, our meta-analysis strengthened the hypothesis of the AD neurogenerative process triggered by OSA. We found decreased Aβ42 levels and increased t-tau levels in the CSF, and increased Aβ burden tested by PET imaging in OSA patients. Besides, changes in Aβ42 level were more pronounced in moderated and severe OSA groups. In clinical practice, our study indicated that existing therapies reducing the severity of OSA could relieve Aβ and tau burden and further delay the progression to MCI or dementia.

## Data availability statement

The original contributions presented in this study are included in the article/supplementary material, further inquiries can be directed to the corresponding author.

## Author contributions

WC and JF contributed to the conception and design of the study. WC and ZD performed literature screening and scoring. WC and ZL performed the statistical analysis. WC wrote the first draft of the manuscript. All authors wrote sections of the manuscript, contributed to manuscript revision, read, and approved the submitted version.

## Appendix

**APPENDIX A:** Search terms used for each database.

Database	Search terms
PubMed	((((Obstructive Sleep Apnea*[Title/Abstract]) OR (sleep disordered breathing [Title/Abstract])) OR (Sleep Apnea*[Title/Abstract])) OR (Upper Airway Resistance [Title/Abstract])) AND ((tau[Title/Abstract]) OR (amyloid beta[Title/Abstract]) OR (amyloid*[Title/Abstract]) OR (PET[Title/Abstract]) OR (Abeta[Title/Abstract]) OR (cerebrospinal fluid[Title/Abstract]))
Embase	1. ‘Obstructive Sleep Apnea’/exp 2. ‘Obstructive Sleep Apnea*’:ab,ti OR ‘sleep disordered breathing’:ab,ti OR ‘Sleep Apnea*’:ab,ti OR ‘Upper Airway Resistance’:ab,ti 3.#1 OR #2 4. ‘tau’: ab,ti OR ‘amyloid*’:ab,ti OR ‘cerebrospinal fluid’:ab,ti OR ‘PET’:ab,ti 5.#3 AND #4 6.[conference review]/lim OR [conference Abstract]/lim OR [conference Paper]/lim OR [editorial]/lim OR [erratum]/lim OR [letter]/lim 7.#5 NOT#6
Web of Science	(TS = (tau OR amyloid OR PET OR cerebrospinal fluid))AND (TS = (Obstructive Sleep Apnea OR OSAHS OR Sleep Apnea OR Upper Airway Resistance))
The Cochrane Library	“Obstructive Sleep Apnea*” OR “sleep disorded breathing” OR “Sleep Apnea*” AND “tau” OR “amyloid*” OR “PET” OR “Abeta” OR “cerebrospinal fluid”

**APPENDIX B:** Quality assessment of included studies using the Newcastle–Ottawa scale score.

Study	S1	S2	S3	S4	C1	E1	E2	E3	Total
Osorio et al., 2014	★	★	−	★	★★	★	★	★	8
Ju et al., 2016	−	★	★	★	★★	★	★	★	8
Liguori et al., 2017	★	★	−	★	★★	★	★	★	8
Yun et al., 2017	★	−	−	★	★★	★	★	★	7
Sharma et al., 2018	★	★	−	★	★★	★	★	★	8
Elias et al., 2018	★	−	★	★	★	★	★	★	7
Liguori et al., 2019	★	★	−	★	−	★	★	★	6
André et al., 2020	★	★	−	★	★★	★	★	★	8
Jackson et al., 2020	−	−	★	★	★★	★	★	★	7
Diáz-Román et al., 2021	−	★	−	★	★	★	★	★	6
Fernandes et al., 2022	★	★	−	★	★★	★	★	★	9

Each study can be awarded a maximum of one star for each item within the Selection (S) and Exposure (E) categories, and a maximum of two stars can be given for Comparability (C) group. S1 = adequate case definition; S2 = representativeness of the cases; S3 = selection of controls; S4 = definition of controls; C1 = comparability of cases and controls; E1 = ascertainment of exposure; E2 = same method of ascertainment for cases and controls; E3 = non-response rate.
